# Isochromosome 13 in a patient with childhood-onset schizophrenia, ADHD, and motor tic disorder

**DOI:** 10.1186/1755-8166-5-2

**Published:** 2012-01-03

**Authors:** Sharon L Graw, Karen Swisshelm, Kirsten Floyd, Billie J Carstens, Marianne Z Wamboldt, Randall G Ross, Sherry Leonard

**Affiliations:** 1 Department of Psychiatry, University of Colorado School of Medicine, Aurora, CO, USA; 2 Colorado Genetics Laboratory, Department of Pathology, University of Colorado School of Medicine, Aurora, CO, USA; 3 Department of Psychiatry and Behavioral Science, Children's Hospital Colorado, Aurora, CO, USA; 4 Research Division, Veterans Affairs Medical Research Service, Denver, CO, USA

**Keywords:** Attention deficit hyperactivity disorder, Chromosome 13, Isochromosome, Schizophrenia, childhood

## Abstract

**Background:**

A small percentage of all cases of schizophrenia have a childhood onset. The impact on the individual and family can be devastating. We report the results of genetic analyses from a patient with onset of visual hallucinations at 5 years, and a subsequent diagnosis at 9 years of schizophrenia, attention deficit hyperactivity disorder (ADHD) with hyperactivity and impulsivity, and chronic motor tic disorder.

**Results:**

Karyotypic analysis found 45,XX,i(13)(q10) in all cells examined. Alpha satellite FISH of isochromosome 13 revealed a large unsplit centromeric region, interpreted as two centromeres separated by minimal or undetectable short-arm material or as a single monocentric centromere, indicating that the isochromosome likely formed post-zygotically by a short arm U-type or centromeric exchange. Characterization of chromosome 13 simple tandem repeats and Affymetrix whole-genome 6.0 SNP array hybridization found homozygosity for all markers, and the presence of only a single paternal allele in informative markers, consistent with an isodisomic isochromosome of paternal origin. Analysis of two chromosome 13 schizophrenia candidate genes, D-amino acid oxidase activator (*DAOA*) and 5-hydroxytryptamine (serotonin) receptor 2A (*5-HTR2A*), failed to identify non-synonymous coding mutations but did identify homozygous risk polymorphisms.

**Conclusions:**

We report a female patient with childhood-onset schizophrenia, ADHD, and motor tic disorder associated with an isodisomic isochromosome 13 of paternal origin and a 45,XX,i(13)(q10q10) karyotype. We examined two potential mechanisms to explain chromosome 13 involvement in the patient's pathology, including reduction to homozygosity of a paternal mutation and reduction to homozygosity of a paternal copy number variation, but were unable to identify any overtly pathogenic abnormality. Future studies may consider whether epigenetic mechanisms resulting from uniparental disomy (UPD) and the lack of chromosome 13 maternal alleles lead to the patient's features.

## Background

Schizophrenia affects approximately 1% of the general population [1]. While symptoms of the disorder generally manifest themselves in the late teens or twenties, they appear before the age of 18 years in approximately 4% of cases [2] and have been reported as early as 3 years [3]. Features of childhood-onset schizophrenia are typically more severe than found in the adult-onset form of the disease, and affected children exhibit auditory hallucinations and delusions, accompanied by a flattened affect, deficits of speech and language, motor development, attention and memory, and social fluency as well as reduced cognitive performance [4]. Long-term outcomes for children with schizophrenia may be worse than adult-onset cases, with continued risk of morbidity [4].

Adult-onset schizophrenia is considered a neurodevelopmental disorder resulting from the interaction of a genetic predisposition with environmental factors. Considerable effort has been put forth to identify genes associated with schizophrenia, and multiple predisposing genes have been identified, including *CHRNA7 *[5], *DAOA *[6], *COMT, DTNBP1, NRG1, DISC1, GRM3*, and *PRODH *[7]. In the last several years, Genome-Wide Association Studies (GWAS) have led to many newly identified schizophrenia susceptibility loci, including the major histocompatibility complex, *CACNA1C*, the *ITIH3-ITIH4 *region, *ZNF804A, PLAA, ANK3*, and others [8-21]. Challenges to these studies include modest effects of individual variations, as well as lack of interstudy reproducibility. Genomic copy number variation (CNV) studies have revealed several regions of duplication/deletion, including a recurrent deletion of 22q11.2 and microdeletions of 1q21.1, 15q11.2, 15q13.3, and a microduplication of 16p11.2 [22-25], with most CNVs having a spectrum of pathologic features and showing evidence of overlap with other forms of brain disorder. Environmental factors that are associated with an increased risk of developing schizophrenia include exposure to stress *in utero *[26], low socioeconomic status [27], urbanicity [28], and cannabis use [29].

Only a few genetic studies have been undertaken to specifically identify genes leading to the development and pathology of childhood-onset schizophrenia. Associations have been reported with *DAOA *[30], *NRG1 *[31], *DTNBP1 *[32,33], and *GAD1 *[34]. Addington and Rapoport [35] found that approximately 10% of childhood-onset schizophrenia patients in their cohort had cytogenetic abnormalities, including deletions of 22q11, atypical/mosaic 45,X and 47,XXX, recurrent duplications of 16p11.2 and *MYT1L *(mapped to 2p25.3), and deletion of *NRXN1 *(on 2p16.3), all of which have been reported in adult-onset schizophrenia patients. Thus, there appears to be considerable overlap of purported candidate regions between adult and childhood-onset schizophrenia, indicating the potential perturbation of similar developmental pathways.

Here we report on a patient with onset schizophrenia symptoms at age 5 years, and a subsequent diagnosis at 9 years of childhood-onset schizophrenia, attention deficit hyperactivity disorder (ADHD) with hyperactivity and impulsivity, and chronic motor tic disorder. Cytogenetic and molecular analyses demonstrate that this patient carries an isodisomic isochromosome 13 of paternal origin.

## Case Presentation

The female patient presented to the study team at 9 years of age. Diagnoses were best estimate diagnoses based on DSM-IV [1], determined after completion of a semi-structured diagnostic interview of both the parents and child [36]. Using a non-hierarchical diagnostic approach, the subject met diagnostic criteria for schizophrenia, attention deficit hyperactivity disorder (hyperactivity/impulsivity subtype), and chronic motor tic disorder. Pregnancy history was unremarkable, and delivery was at term via planned caesarean section secondary to breach presentation. Early milestones were delayed, and the patient underwent a developmental evaluation at 2 1/2 year of age. Academically, the patient has repeated grades and is currently in a special needs program with some mainstreamed class time.

The patient first reported visual hallucinations at 5 years, and by 7 years psychotic symptoms included frequent visual hallucinations, command auditory hallucinations, and grandiose beliefs about superhuman powers. Patient symptoms described by the parents included flat affect, periods of incoherence, and loosening of associations. All symptoms were present consistently for 2 1/2 years prior to study enrollment. The patient is under treatment with risperidone and amantadine and is able to manage behavior during the day. Her attention span is reported to be difficult but improved with treatment. She still experiences auditory hallucinations which are now ego dystonic and identifiable. In addition, the patient has underdeveloped social skills and has developed relapsing and remitting vocal tics, as well as simple and complex motor tics.

Family history includes a maternal great grandmother with recurrent psychiatric inpatient admissions, but insufficient information was available for the study team to clarify diagnosis. The patient's mother had a history of depressive symptoms.

## Results

### Cytogenetics

**Figure 1:**
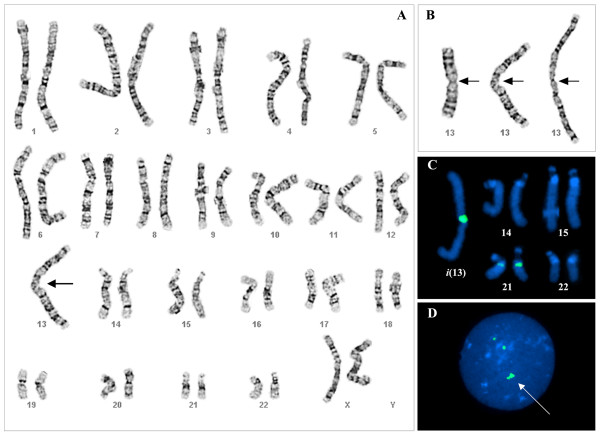
**Cytogenetic analysis of patient**. A) 45,XX,i(13)(q10) chromosomal karyotype of proband, showing the presence of a single metacentric chromosome consisting of two copies of the patient's chromosome 13 joined at the centromere (arrow), B) Composite of derivative 13 homologues from three metaphase spreads with centromeres indicated by arrows, C) Composite of acrocentric homologues hybridized with alpha satellite DNA probe for centromeres of chromosomes 13 and 21, demonstrating the presence of a large centromere on the isochromosome 13, D) Interphase cell with alpha satellite DNA probe for centromeres of chromosomes 13 and 21. Arrow points to large likely signal of the isochromosome 13.

Karyotypic analysis of twenty G-banded metaphases from mitogen stimulated peripheral blood lymphocytes was performed at the 450-650 band level. Analysis revealed 45 chromosomes in each cell examined, with a Robertsonian-type translocation of the long arms of each homologue of chromosome 13 (Figures 1a and 1b). The patient initially had a reported karyotype of 45,XX,der(13;13)(q10;q10) which subsequently was modified to 45,XX,i(13)(q10) upon molecular characterization.

### Fluorescence *In Situ *Hybridization (FISH)

Centromere status was determined by FISH analysis with an alpha satellite probe homologous to the centromeric regions of chromosomes 13 and 21 (Figures 1c and 1d) which showed the presence of a large, chromosome 13 centromere. This was interpreted as either two centromeres separated by minimal or undetectable short-arm material or as a single, large chromosome 13 centromere. Paternal and maternal cytogenetic analyses revealed normal karyotypes.

Since there is a reported association between schizophrenia and deletions in chromosome 22q11.2 [37], this region underwent examination in the patient and her parents. FISH analysis in the DiGeorge/Velocardiofacial syndrome critical region at chromosome 22q11.2 with the DiGeorge/VCFS Tuple1 probe did not identify any deletion or duplication in the patient or her parents in this region on chromosome 22.

### Simple tandem repeats

Because of the initial finding of the patient's der(13;13)(q10;q10), the chromosome 13 content of the patient and her parents underwent characterization with twelve simple tandem repeats (STRs) spanning chromosome 13 plus a control STR from chromosome 21. The patient was homozygous for all chromosome 13 STRs (Table 1). In six STRs, the patient had only paternal alleles, with lack of detectable maternal alleles. Five of these STRs were completely informative, the patient inheriting only a single paternal allele when the father was heterozygous. Based on these STRs, we were able to conclude that the patient carried alleles from only a single paternal chromosome 13, and thus had an isodisomic isochromosome 13 accompanied by a karyotype of 45,XX,i(13)(q10). The remaining six STRs of the twelve examined were less informative, and while the patient was homozygous at these STRs, the pattern was consistent with either paternal or maternal inheritance.

**Table 1:** STR analysis of patient and her parents

Marker	Position	Subject	Genotype	Inheritance
D13S175	1,653,855	Patient	1/1	**Homozygous, P**
		Father	1/2	
		Mother	3/4	
				
D13S171	14,064,951	Patient	1/1	Homozygous, M or P
		Father	1/1	
		Mother	1/2	
				
D13S218	19,831,225	Patient	1/1	Homozygous, M or P
		Father	1/2	
		Mother	1/1	
				
D13S155	34,882,886	Patient	1/1	Homozygous, M or P
		Father	1/2	
		Mother	1/3	
				
D13S176	41,205,385	Patient	1/1	**Homozygous, P**
		Father	1/2	
		Mother	1/3	
				
D13S71	75,575,535	Patient	1/1	Homozygous, P
		Father	1/1	
		Mother	2/2	
				
D13S154	76,860,260	Patient	1/1	**Homozygous, P**
		Father	1/2	
		Mother	3/4	
				
D13S174	102,954,076	Patient	1/1	Homozygous, M or P
		Father	1/2	
		Mother	1/3	
				
D13S1809	106,132,754	Patient	1/1	**Homozygous, P**
		Father	1/2	
		Mother	2/3	
				
D13S173	107,806,947	Patient	1/1	Homozygous, M or P
		Father	1/2	
		Mother	1/1	
				
D13S1265	109,328,688	Patient	1/1	**Homozygous, P**
		Father	1/2	
		Mother	2/3	
				
D13S1295	113,094,472	Patient	1/1	Homozygous, M or P
		Father	1/2	
		Mother	1/1	
				
D21S270		Patient	1/2	
		Father	1/2	
		Mother	2/3	

Chromosome 13 and 21 markers, along with position on chromosome (ensembl.org). Alleles were assigned arbitrary numbers, and genotype information is given for the patient and parents. Inheritance patterns have been determined. P indicates paternal inheritance and M indicates maternal inheritance. **Bolded **inheritance patterns demonstrate the expression of a single paternal allele and no maternal alleles in the patient, when the father is heterozygous.

### Affymetrix Whole-Genome Human SNP 6.0 microarrays

**Figure 2:**
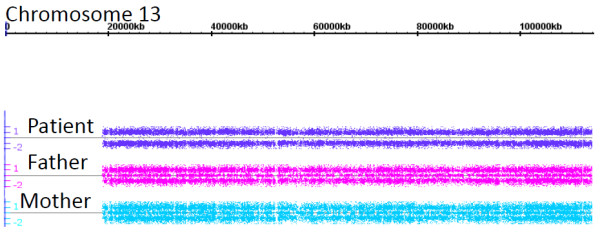
**Chromosome 13 Affymetrix Whole-Genome Human SNP 6.0 analysis**. Allele analysis demonstrates complete homozygosity of the patient's chromosome 13, with an absence of heterozygous alleles. The patient's parents have both homozygous and heterozygous alleles. Heterozygous markers lie along the center line, while homozygous markers fall either above or below the line.

In order to further refine the patient's chromosome 13 status, DNA from the patient and her parents were utilized for Affymetrix whole genome Human SNP 6.0 microarray evaluation. Allele analysis demonstrated that the patient was homozygous for all of chromosome 13 (Figure 2). In contrast, both paternal and maternal DNA demonstrated chromosome 13 heterozygosity. Chromosome 13 CNV status was examined, and the patient was found to have minimal evidence of deletions or duplications [Additional File 1]. Of the nine regions of duplication/deletion that were identified on chromosome 13, none reached default criteria required for identification as a CNV segment (Minimum 100,000 bp or 50 markers for gain/loss) with any degree of certainty. Five were not located in any gene, and four were located in genes (*ATP8A2, LHFP, PHF11, RCBTB1*, and *PIBF1 *(C13orf24)) not yet shown to be associated with schizophrenia. Thus, of the five chromosome 13 genes with evidence of duplication or deletion in the patient, none appears to be candidates for her pathology. While not currently associated with schizophrenia, these genes may be investigated in future studies of this patient.

**Figure 3:**
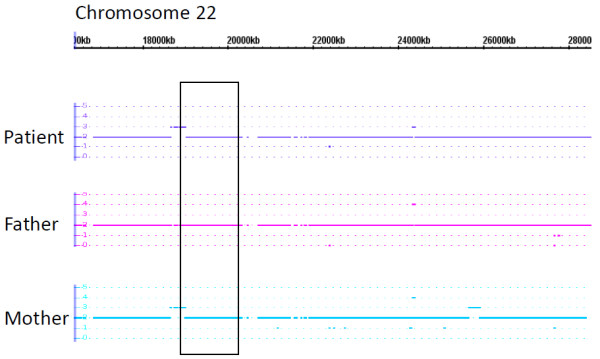
**Chromosome 22 Affymetrix Whole-Genome Human SNP 6.0 analysis**. The 1.5 Mb region of chromosome 22 associated with 22q11.2 Deletion Syndrome in schizophrenia [85] is noted by a box. The patient and her mother both contain a small duplication at the centromeric end of the 22q11.2 deletion syndrome region, but neither has a deletion.

**Figure 4:**
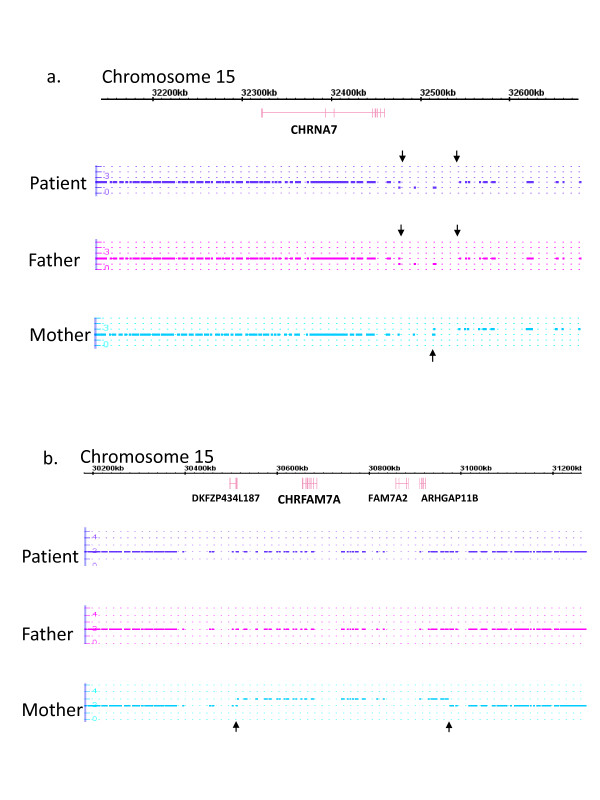
**Chromosome 15 Affymetrix Whole-Genome Human SNP 6.0 analysis**. A) Copy number analysis flanking *CHRNA7 *gene, with downward arrows indicating a deleted region in the patient and father just distal to *CHRNA7 *and upward arrows indicating the beginning of a duplicated region in mother. B) Copy number analysis flanking *CHRFAM7A *gene, with arrows indicating the beginning and the end of the duplicated region in the patient's mother.

Several other regions of the genome associated with CNVs in schizophrenia were examined, including chromosome 22 (Figure 3), which is implicated in childhood onset schizophrenia [35], and chromosome 15 (Figure 4), demonstrated to have copy number variations in patients with schizophrenia [22]. While the patient and her mother did have a small duplication on chromosome 22 (Figure 3), there was no evidence of deletions in the region implicated in 22q11.2 Deletion Syndrome. The patient and her father both had a deletion on chromosome 15 distal to the *CHRNA7 *gene, encoding the α7* nicotinic receptor (Figure 4a). The patient's mother had a chromosome 15 duplication of the *CHRFAM7A *gene (Figure 4b), which is also associated with schizophrenia [38,39] and modulates α7* nicotinic receptor activity [40,41]. While it is possible that the patient's prenatal environment contained perturbations in nicotinic receptor activity, it seems unlikely that chromosome 15 CNVs in this family can account for the patient's severe pathology. No CNVs were found in the regions encoding *NRXN1 *and *MYT1L *on chromosome 2 [Additional file 2]. Analysis of chromosome 16p11.2 in our patient reveals no significant regions of duplication or deletion. However, the proband and her parents contain regions of LOH on chromosome 16p11.2 [Additional file 3]. Since the patient's chromosome 16 LOH is recapitulated in her parents, it is unlikely to be involved in the patient's pathology.

### Gene Analysis

Multiple genes encoded by chromosome 13 have been suggested to contribute to schizophrenia susceptibility, including *DAOA *[42], *5-HTR2A *[43], *KPNA3 *and *KPNB3 *[44], *ESD *[45], *ATXN8OS *[46], *KFL5 *[47], *EFNB2 *[48], and *PCDH8 *[49]. Since it was not possible to narrow down the candidate region of chromosome 13, two genes were chosen for sequence analysis based on their robust association with schizophrenia: *DAOA *[42] and *5-HTR2A *[43]. Protein coding sequence from these two genes, along with 300 bp of upstream non-coding sequence, 300 bp downstream, and at least 25 bp into the splice junctions, were sequenced in patient DNA and compared to canonical sequence (ensemble.org release 61). Five SNPs were identified in the patient, two (rs6313 and rs6311) in *5-HTR2A *and three (rs1361562, rs2391191, and rs778294) in *DAOA *[Additional file 4]. In all cases the patient was homozygous for the detected polymorphism, consistent with isodisomy. The patient's father also was found to be homozygous for the *5-HTR2A *SNPs rs6313 and rs6311, which are in linkage disequilibrium and are associated with schizophrenia [43]. None of the polymorphisms identified in the patient was novel, and all had been previously described (ensembl.org and ncbi.nlm.nih.gov). None of the polymorphisms noted in the coding regions of either gene was non-synonymous.

## Conclusions

This report describes a patient with isochromosome 13, accompanied by childhood-onset schizophrenia, ADHD with hyperactivity and impulsivity, and chronic motor tic disorder. Cytogenetic analysis revealed that the proband carries a 45,XX,i(13)(q10) karyotype, and additional marker analysis demonstrated that the derivative 13 chromosome was a paternally derived isodisomic isochromosome. Since the patient's isochromosome 13 had no evidence of recombination, it most likely formed post-zygotically by monosomy rescue [50]. However, we cannot rule out the possibility that the proband's father is a mosaic carrier of the isochromosome 13. Unless associated with trisomy 13, cases of isochromosome 13 typically do not lead to pathology, although the long-term follow-up needed for detection of mental illness has not typically been reported [51-53]. There are several potential mechanisms by which the proband's isochromosome 13 could lead to the observed pathology. We were able to examine two of these: reduction to homozygosity of a paternal mutation and reduction to homozygosity of a paternal CNV. The failure to demonstrate either of these leads us to consider whether alternative mechanisms, such as epigenetic regulation, may be responsible for the patient's features.

We initially hypothesized that the patient's father was a heterozygous carrier of a mutation on chromosome 13 that could lead to a phenotype when homozygous in the patient. No overtly pathogenic mutations were detected, although the patient and her father were shown to be homozygous carriers of *5-HTR2A *polymorphisms associated with predisposition to schizophrenia (discussed below). We also examined whether the patient exhibited a reduction to homozygosity of a heterozygous chromosome 13 paternal CNV. While several of these small regions were associated with genes, none of these genes is known to be associated with schizophrenia. Thus, chromosome 13 CNVs do not appear to be a robust candidate for her pathology.

We investigated other non-chromosome 13 CNVs and cytogenetic abnormalities. Childhood-onset schizophrenia abnormalities identified by Addington and Rapoport [35] include 22q11, 45,X atypical/mosaic, 47,XXX, duplications of 16p11.2, *MYT1L *duplication on chromosome 2, and *NRXN1 *deletion on chromosome 2. Affymetrix SNP analysis and standard cytogenetics allow us to conclude that this patient has no evidence of the pathologic alterations described by Addington and Rapoport. Similarly, while there was evidence of CNVs on chromosome 15 in the regions associated with schizophrenia, it seems unlikely that chromosome 15 CNVs can account for her severe pathology.

Schizophrenia has been reported in several patients with chromosome 13 rearrangements, and these cases illustrate the difficulty in establishing a causal rather than coincidental relationship between the cytogenetic abnormality and psychiatric pathology. Roberts and coworkers [54] reported an inversion/insertion involving chromosome 13, with breakpoints at 13q21.3, 13q32, and 13q31, and although the abnormality was found in several family members with "mental subnormality, personality defects and frank psychosis," concordance was not 100%. In this case, pathology may be the result of breakpoint disruption of genes on chromosome 13. Itokawa and coworkers [55] reported a karyotype of 46,XY,t(4;13)(p16.1;q21.31) in a single patient with a diagnosis of paranoid schizophrenia, and pathology may be associated with disruption of genes on either chromosome 4 or 13. In contrast, schizophrenia in patients with balanced t(13;14), which are common and heterologous, has been reported [56-58], and it is likely that the occurrence of both a chromosome translocation and psychiatric pathology in these patients is coincidental. Thus, while chromosome 13 abnormalities have been found in patients with schizophrenia, it has proven difficult to demonstrate unambiguously that the chromosome abnormality leads to the pathology.

The results presented here lead us to question whether the patient's isochromosome 13 directly leads to her symptoms or is a coincidental finding. Isochromosome 13 is rare [59] and the occurrence of both i(13)(q10) and childhood onset schizophrenia in a single individual would be extremely unlikely. While we have investigated two potential mechanisms by which i(13)(q10) could lead to the patient's pathology, our investigations have not been exhaustive. Mutations in chromosome 13 genes not studied, or in parts of *5-HTR2A *or *DAOA *not sequenced here, may be pathogenic.

There are also additional mechanisms that may be considered. For example, there has been intense speculation concerning the role of epigenetic modification in the susceptibility and development of schizophrenia [60-64]. In cases where the origins have been determined, both maternally and paternally derived uniparental disomy (UPD) of chromosome 13 have been associated with normal development [51-53]. In many cases, however, follow-up has not been extended past childhood, so the possibility of later development of psychiatric pathology cannot be ruled out. There is evidence that regions of chromosome 13 are imprinted in an individual, tissue-specific, or polymorphic fashion, and that regulation may be complex. For example, studies demonstrate allele-specific and parent-of-origin-specific differential expression of *5-HTR2A *[65-71] and Abdolmaleky and coworkers [72] have found evidence for differences between epigenetic regulation of *5-HTR2A *between patients with schizophrenia or bipolar disorder and controls.

There is precedence for the co-occurrence of uniparental disomy (UPD) and childhood-onset schizophrenia. Seal and coworkers [73] reported a case of childhood-onset schizophrenia associated with segmental UPD of chromosome 5q32-qter. Linkage of schizophrenia to chromosome 5p23.2-p24 has been previously described by multiple groups [74-80], and several candidate genes have been proposed, including the *GABA *receptor genes, *EPN4 *[81], *SMAD5*, and *SPRY4 *[82].

The patient described here exhibits childhood-onset schizophrenia, ADHD, and chronic motor tic disorder accompanied by the karyotype 45,XX,i(13)(q10q10). Although we did not find overtly pathogenic chromosome 13 CNVs or mutations in the patient, there may be unidentified mutations in regions of chromosome 13 that were not examined. We did demonstrate that the patient and her father are homozygous carriers of 5-*HTR2A *SNPs associated with schizophrenia. Considering the complete homozygosity and UPD of the patient's chromosome 13, epigenetic modifications should be investigated as a potential explanation for the patient's findings.

## Methods

### Patient Recruitment

The patient originally was identified requiring treatment for psychosis, and patient and family material and information were collected as part of an ongoing study which includes cytogenetic analysis of children with psychotic symptoms. Informed consent and assent were obtained as monitored by a local Institutional Review Board.

### Cytogenetics

Cytogenetic analysis of peripheral blood lymphocytes was conducted by the Colorado Genetics Laboratory using standard techniques. Following two days in RPMI/10% fetal bovine serum, cultures were exposed to a final concentration of 0.06 micrograms/mL of Colcemid (Irvine Scientific, Santa Ana, CA) for 30 min - 2 hr in order to capture metaphase cells. Cells were exposed to 0.075 M KCl hypotonic for 30 min and fixed in 3:1 methanol:glacial acetic acid. Trypsin G-banding was performed following standard procedures [83].

### Fluorescence *in situ *hybridization (FISH)

FISH analysis utilized an alpha satellite probe with homology to the centromeric heterochromatic regions of chromosomes 13 and 21. FISH analysis for the DiGeorge/Velocardiofacial syndrome (VCSF) critical region at chromosome 22q11.2 was performed with the DiGeorge/VCFS Tuple1 probe using standard methods (Abbott Molecular, Abbott Park, Illinois).

### Simple Tandem Repeats (STRs)

Polymerase Chain Reaction was performed with *Taq *Gold (Applied Biosystems, Foster City, CA) using primers designed from UniSTS http://ncbi.nlm.nih.gov/unists on a GeneAmp 9700 thermal cycler (Applied Biosystems, Inc., Foster City, CA). Products were analyzed on an Applied Biosystem's 3100 Avant Genetic Analyzer, and results interpreted with GeneMapper version 3.5 (Applied Biosystem, Inc.). DNA was purified by phenol extraction from lymphoblasts using standard protocols [84].

### Affymetrix GenomeWide 6.0 SNP microarrays

The Affymetrix Genome-Wide Human SNP 6.0 assay was performed according to the manufacturer's instructions (Affymetrix, Santa Clara, CA) in the Genomics and Microarray Core Laboratory at the University of Colorado School of Medicine. Data were combined with HapMap samples provided by Affymetrix and analyzed using the Affymetric Genotyping Console version 4.0. Algorithms for copy number calling are based on the Hidden-Markoff Model, where copy number patterns of neighboring markers are taken into account.

### Gene Sequence Analysis

Genomic sequences and intron/exon structure for D-amino acid oxidase activator (*DAOA*; ENSG00000192346) and 5-hydroxytryptamine (serotonin) receptor 2A (*5-HTR2A*; ENSG00000102468) were taken from Ensembl Release 61 - Feb 2011 and earlier releases http://ensembl.org, and were used to generate primers for PCR and sequencing with the program Primer3 http://frodo.wi.mit.edu/. Products were sequenced directly without additional purification using the BigDye^® ^Terminator v3.1 Cycle Sequence Kit (Applied Biosystems, Austin, TX) on an ABI 3100-Avant Genetic Analyzer (Applied Biosystems, Inc.), and analyzed with Sequencher 4.8 (Gene Codes Corporation, Ann Arbor MI). Subject sequences were compared to database sequences using Basic Local Alignment Search Tool (BLAST) http://www.ncbi.nlm.nih.gov/BLAST/.

### Consent

Written informed consent was obtained from the patient's parents for publication of this Case Report. A copy of the written consent is available for review by the Editor-in-Chief of this journal.

## List of abbreviations

ADHD: attention deficit hyperactivity disorder; *ANK3*: ankyrin 3; *ATP8A2*: ATPase, class I, type 8A, member 2; BLAST: Basic local alignment search tool; bp: base pair; *CACNA1C*: calcium channel, voltage-dependent, L type, Alpha-1C subunit; *CHRFAM7A*: *CHRNA7 *(cholinergic receptor, nicotinic, alpha 7, exons 5-10) and *FAM7A *(family with sequence similarity 7A, exons A-E) fusion; *CHRNA7*: cholinergic receptor, nicotinic, alpha 7; CNV: copy number variation; *COMT*: catechol-O-methyltransferase; *DAOA*: D-amino acid oxidase activator; *DISC1*: disrupted in schizophrenia 1; DSM-IV: Diagnostic and Statistical Manual of Mental Disorders, 4^th ^edition; *DTNBP1*: dystrobrevin binding protein 1; *EPN4*: epsin 4; FISH: fluorescence *in situ *hybridization; GABA: gamma-aminobutyric acid; *GAD1*: glutamate decarboxylase 1; *GRM3*: glutamate receptor, metabotropic 3; *HTR2A*: 5-hydroxytryptamine (serotonin) receptor 2A; *ITIH3*: inter-alpha-trypsin inhibitor, heavy chain 3; ITIH4: inter-alpha-trypsin inhibitor, heavy chain 4; *LHFP*: lipoma HMGIC fusion partner; LOH: loss of heterozygosity; *MYT1L*: myelin transcription factor 1-like; *NRG1*: neuregulin 1; *NRXN1*: neurexin 1; PCR: polymerase chain reaction; *PHF11*: PHD finger protein 11; *PIBF1*: C13orf24; progesterone-induced blocking factor 1; *PLAA *: phospholipase A2-activating protein; *PRODH*: proline dehydrogenase (oxidase) 2; *RCBTB1*: Regulator of chromosome condensation (*RCC1*) and BTB (POZ) domain contain protein 1; *SMAD5*: mothers against decapentaplegic, drosophila, homolog of, 5; SNP: single nucleotide polymorphism; *SPRY4*: sprout, drosophila, homolog of, 4; STR: simple tandem repeat; UPD: uniparental disomy; UTR: untranslated region; VCFS: velocardiofacial syndrome; *ZNF804A*: zinc finger protein 804A

## Competing interests

The authors declare that they have no competing interests.

## Authors' contributions

SG conceived of the study and its design, performed the sequencing, and analyzed the data, and wrote the manuscript. KS supervised the cytogenetic analysis. KF performed the STR analysis. BJC performed the cytogenetic analysis. MZW and RGR examined the patient and provided clinical details. SL participated in study design and data analysis. All authors read and approved the final manuscript.

## Supplementary Material

Additional file 1 **Chromosome 13 CNV analysis of patient and father**. CNV analysis indicates the presence of two copies of almost all chromosome 13 markers in the patient and her father. Click here for file

Additional file 2 **Affymetrix Whole-Genome Human SNP6.0 CNV analysis of *MYT1L *and *NRXN1 *genes**. Analysis indicates that the patient and her parents have two copies of the *MYT1L *and *NRXN1 *genes. Click here for file

Additional file 3 **Affymetrix Whole-genome Human SNP6.0 analysis of chromosome 16**. Analysis indicates that the patient and her parents have regions on chromosome 16 with reduced heterozygosity, and that no significant deletions or duplications are found. Click here for file

Additional file 4 **SNP analysis**. SNPS were detected in *DAOA *and *HTR2A *genes of patient. Click here for file
