# Supplementary material for: Isochromosome 13 in a patient with childhood-onset schizophrenia, ADHD, and motor tic disorder
Source: Mol Cytogenet. 2012 Jan 3;5:2. doi: 10.1186/1755-8166-5-2 (PMC3274485; doi:10.1186/1755-8166-5-2)

Additional File 1. Chromosome 13 CNV analysis of patient's and her father's DNA indicating the presence of two copies of almost all chromosome 13 markers. Copy number is given on left (from 0 to 4). Results from the patient are shown in purple and her father in blue. None of the small blocks indicating deviation from a copy number of two reached default criteria for identification as a CNV segment.

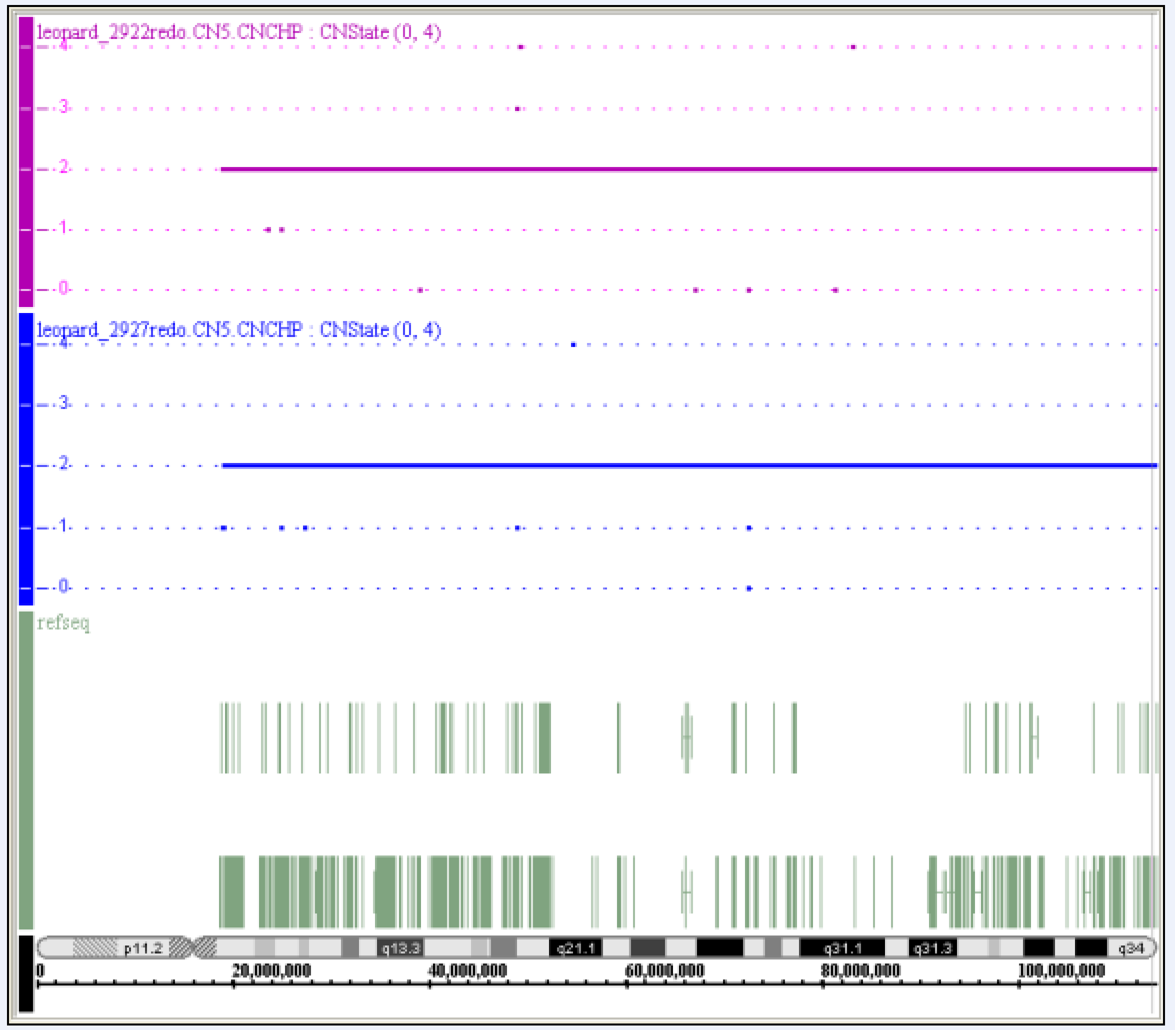

Supplement: Additional file 1 — Chromosome 13 CNV analysis of patient and father. CNV analysis indicates the presence of two copies of almost all chromosome 13 markers in the patient and her father. [file 1755-8166-5-2-S1.PDF]
