# Supplementary material for: Isochromosome 13 in a patient with childhood-onset schizophrenia, ADHD, and motor tic disorder
Source: Mol Cytogenet. 2012 Jan 3;5:2. doi: 10.1186/1755-8166-5-2 (PMC3274485; doi:10.1186/1755-8166-5-2)

Additional File 2. Affymetrix Whole-Genome Human SNP6.0 CNV analysis of a) *MYT1L* gene at chromosome 2p25.3, and b) *NRXN1* at 2p16.3, indicating that all subjects have 2 copies of both genes, including the patient (purple), her father (blue), and her mother (green). Copy number range is indicated on left side (from 0 to 4), and copy number throughout the region is shown by a heavy colored horizontal line.

a) *MYT1L*

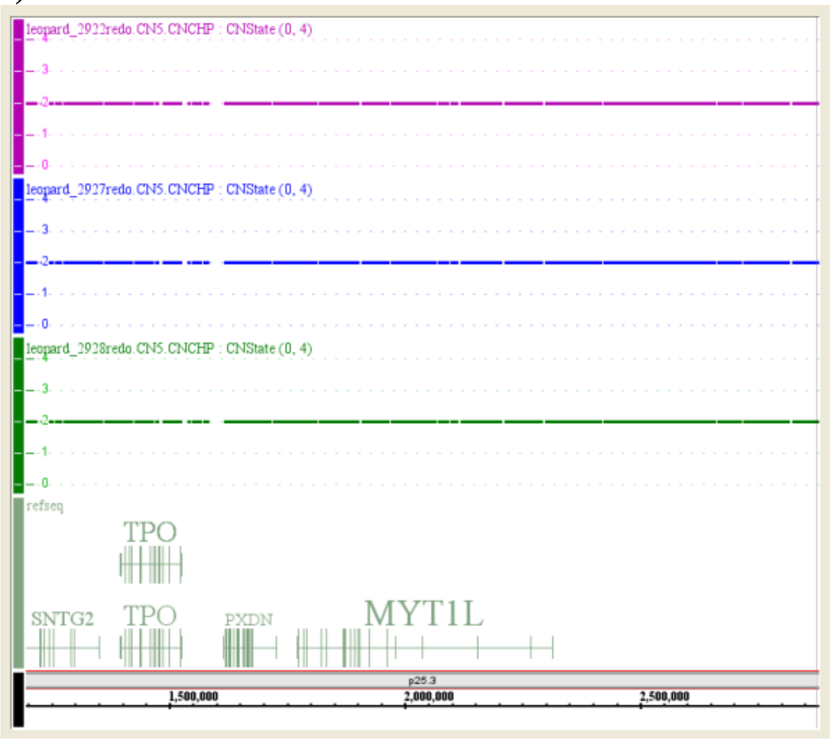

b) *NRXN1*

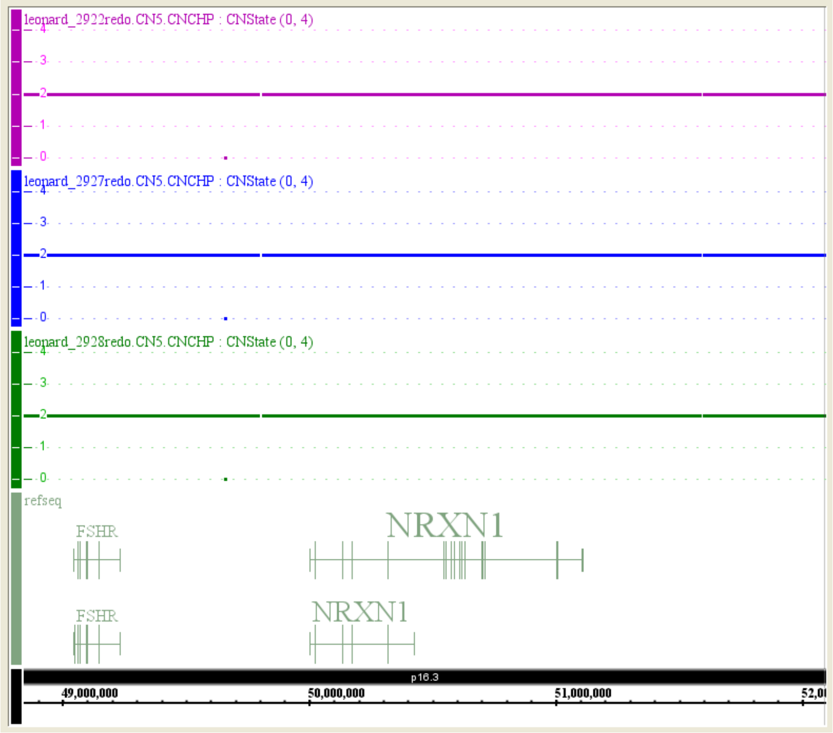

Supplement: Additional file 2 — Affymetrix Whole-Genome Human SNP6.0 CNV analysis of MYT1L and NRXN1 genes. Analysis indicates that the patient and her parents have two copies of the MYT1L and NRXN1 genes. [file 1755-8166-5-2-S2.PDF]
