# Supplementary material for: Isochromosome 13 in a patient with childhood-onset schizophrenia, ADHD, and motor tic disorder
Source: Mol Cytogenet. 2012 Jan 3;5:2. doi: 10.1186/1755-8166-5-2 (PMC3274485; doi:10.1186/1755-8166-5-2)

Additional File 3. Affymetrix Whole-genome Human SNP6.0 analysis of chromosome 16.

a) LOH analysis. The patient (purple) has several regions of reduced heterozygosity (LOH), indicated by the vertical bars, which are found in her father (blue) and mother (green). Regions of the chromosome without LOH are indicated by the horizontal lines. A gap indicates a pericentromeric region not queried by the analysis.

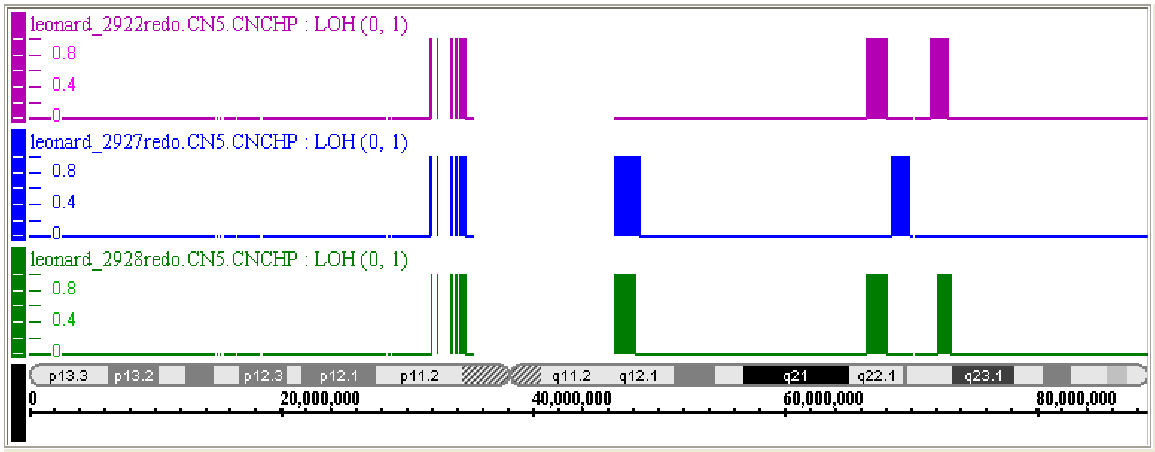

b) CNV analysis. Copy number is indicated on the left, from 0 to 4 copies. The patient (purple) and her parents (father blue, mother green) have no significant deletions or duplications of chromosome 16, as shown by the horizontal lines indicating two copies. A gap indicates a pericentromeric region not queried by the analysis.

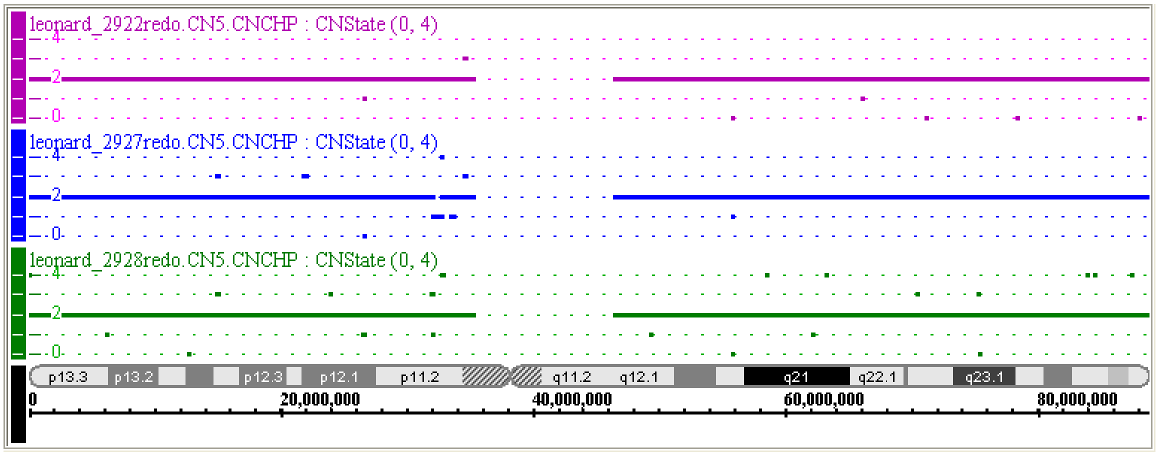

Supplement: Additional file 3 — Affymetrix Whole-genome Human SNP6.0 analysis of chromosome 16. Analysis indicates that the patient and her parents have regions on chromosome 16 with reduced heterozygosity, and that no significant deletions or duplications are found. [file 1755-8166-5-2-S3.PDF]
