# Supplementary material for: Isochromosome 13 in a patient with childhood-onset schizophrenia, ADHD, and motor tic disorder
Source: Mol Cytogenet. 2012 Jan 3;5:2. doi: 10.1186/1755-8166-5-2 (PMC3274485; doi:10.1186/1755-8166-5-2)
Supplement: Additional file 4 — SNP analysis. SNPS were detected in DAOA and HTR2A genes of patient. [file 1755-8166-5-2-S4.PDF]

Additional File 4. SNPs detected in *DAOA* and *HTR2A* genes of patient. SNP indicated by arrow.

DAOA

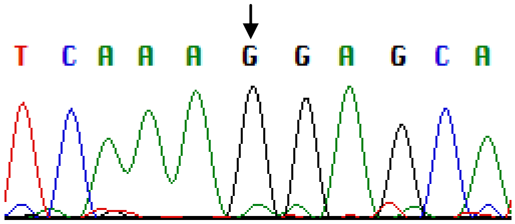

rs2391191

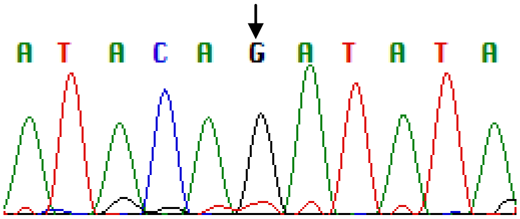

rs1361562

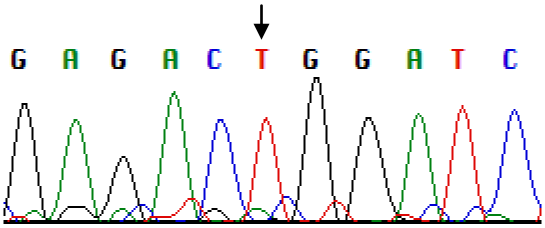

rs778294

HTR2A

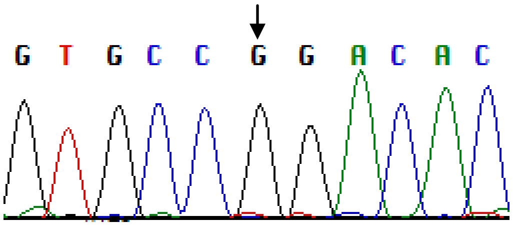

rs6311

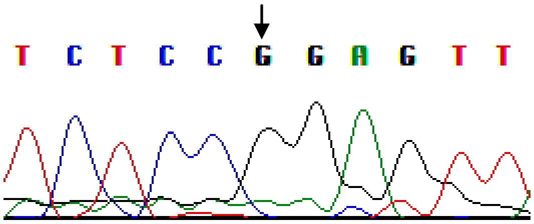

rs6313
